# The Impact of Cooperative Learning on University Students’ Academic Goals

**DOI:** 10.3389/fpsyg.2021.787210

**Published:** 2022-01-05

**Authors:** Santiago Mendo-Lázaro, Benito León-del-Barco, María-Isabel Polo-del-Río, Víctor M. López-Ramos

**Affiliations:** Cooperative Research Laboratory, Universidad de Extremadura, Badajoz, Spain

**Keywords:** cooperative learning, team work, academic goals, university students, quasi-experimental study

## Abstract

Cooperative learning encourages the development of interpersonal skills and motivates students to participate more actively in the teaching and learning process. This study explores the impact of cooperative learning on the academic goals influencing university students’ behavior and leading to the attainment of a series of academic objectives. To this end, a quasi-experimental pretest-posttest control group design was used, with a sample of 509 university students from Preschool, Primary and Social Education undergraduate degree courses. Using the Academic Goals Questionnaire (AGQ), pretest and posttest measures were taken *via* self-reports to evaluate three types of academic goals: learning goals, social reinforcement goals and achievement goals. The results show that cooperative learning is an effective tool for encouraging university students to develop academic goals that motivate them to fully engage with the tasks they are set in order to acquire knowledge and skills (learning goals). In addition, when students are asked to work as part of a team on an autonomous basis without the structure and supervision necessary to ensure a minimum standard of cooperation, they display a greater tendency toward social reinforcement goals than toward learning and achievement goals. These findings contribute new knowledge to the conceptual framework on cooperative learning. Goals may be considered one of the most important variables influencing students’ learning and the use of cooperative learning techniques in university classrooms creates the necessary conditions for encouraging students to develop goals oriented toward learning.

## Introduction

The adaptation of university degrees to the European Higher Education Area led to a shift toward a new paradigm of learning as skills development, lending renewed impetus to methodologies based on active constructive learning such as cooperative learning (Pallisera et al., 2010; Gil, 2015). This methodology enables university students to acquire basic skills and increases their motivation to participate actively in the learning process (Pegalajar and Colmenero, 2013; Fernandez-Rio et al., 2017; Mendo et al., 2018).

Although scholarly interest in this type of methodology has focused largely on primary and secondary education, research into the use of cooperative learning in university settings has grown more common in recent years (Carrasco and Giner, 2011; Barba et al., 2012; Pegalajar and Colmenero, 2013). Team work in university settings was traditionally assumed to be a skill acquired through experience or intuition and groups were frequently set up to encourage learning (Fabra, 1992). These days, learning methods based on cooperation are more and more widely used with the aim of encouraging team work, allowing students to learn to work as part of a team, improving performance and learning and developing interpersonal skills (Gottschall and Garcia-Bayonas, 2008; Gaudet et al., 2010; León et al., 2015; Mendo et al., 2016; Baena-Morales et al., 2020).

University teachers must create an environment that is conducive to effective team learning. This requires effort, interest and recognition of the fact that the proper functioning of the teams and attainment of the learning objectives depend upon the teacher’s role, as does student satisfaction with group work. Cooperative learning is useful in addressing a lack of motivation for learning among students and it is viewed as a positive teaching methodology capable of motivating students in university settings (Ovejero, 1993; van Wyk, 2012; Tran, 2019; Cecchini et al., 2021; Liu and Lipowski, 2021). Slavin (2014) identifies several theoretical perspectives on the effects of cooperative learning, including a motivational perspective emphasizing the incentives it provides for students to complete their academic work. From this perspective, it becomes clear why studying academic motivation in relation to the goals that guide or shape academic activity more generally is so relevant (González, 1997). Academic goals influence students’ behavior in the classroom and drive them to achieve a series of objectives in their academic lives (De la Fuente, 2004).

Research into different types of academic goals has traditionally focused on two categories: learning goals and performance goals. Learning goals, also known as mastery goals and task-focused goals, are related to mastery and enjoyment of a task by students, who are keen to learn and improve their skills. Performance goals, meanwhile, are also known as execution goals or ego-involvement goals and relate to caring for one’s image, achieving an outcome or demonstrating one’s abilities (León et al., 2019; Alhadabi and Karpinski, 2020; Putarek and Pavlin-Bernardić, 2020).

This dichotomous model of academic goals has evolved over time into a trichotomous model (Elliot and Harackiewicz, 1996), then a 2 × 2 model (Elliot and McGregor, 2001; Elliot, 2005), and finally, the current 3 × 2 model (Elliot et al., 2011). In the trichotomous model (Elliot and Harackiewicz, 1996), performance goals are divided into approach goals and avoidance goals (based on the direction of the goal). The 2 × 2 model (Elliot and McGregor, 2001; Elliot, 2005) expands upon the concept of learning goals by dividing them into approach and avoidance goals (based on the orientation of the goal). Finally, the 3 × 2 model (Elliot et al., 2011) seeks to enhance the precision of the 2 × 2 model by incorporating elements such as the task, self and other into evaluations of students’ skills.

When students apply themselves to academic tasks, they can pursue multiple goals resulting from a combination of learning goals and performance goals (Kaplan and Maehr, 2002). These goals are not exclusively personal and are flexible and dynamic, making it possible to influence them within the learning environment (Chiecher, 2017).

Orientation toward one type of goal or another may change over time if university teachers encourage these goals using different methodologies and taking action to motivate students (Andreev et al., 2020). Teaching staff can help students identify and set learning goals, as well as encourage them to adopt a different approach to their goals through the teaching methodology used (Gaeta, 2014; Chiecher, 2017). While completing a task by cooperating with other students may be beneficial for student motivation and learning (Johnson and Johnson, 1999), cooperative learning in a university setting can be an effective method for influencing university students’ academic goals. Formal cooperative learning activities are planned in advance, ensuring that students interact and work together to achieve learning goals and maximizing the development of cognitive and social skills (Johnson and Johnson, 2005).

This study views cooperative learning as an opportunity for encouraging the development of key skills among university students and aims to analyze the impact of cooperative methodology on academic goals (learning goals, social reinforcement goals and achievement goals) among university students on undergraduate degree courses in Education. It seeks to demonstrate the efficacy of cooperative learning in a university setting as a method for fostering and preserving goals that drive university students to improve their skills, learn and enjoy their academic work in the learning environment.

## Materials and Methods

### Participants

The sample for the study comprised 509 students (82% women) on undergraduate degree courses in Preschool Education (PS), Primary Education (PE), and Social Education (SE) at the University of Extremadura (Spain). Their ages ranged from 18 to 34, with a mean age of 20.26 (SD = 2.49) (Table 1).

**TABLE 1:** Distribution of the experimental and control groups.

	Group		Total	
Degree courses	Experimental	Control	*n*	%
Preschool education	122	60	182	35.8
Primary education	128	65	193	37.9
Social education	72	62	134;	26.3
Total	322	187	509	100

The participants in the experimental group were selected on the basis of their enrollment on a module taught by a teacher with specific training in cooperative learning in the second semester of the 2019-2020 academic year. To keep the groups as equivalent as possible, students in the control group were selected from the same degree courses, also in the second semester of the 2019-2020 academic year, but from modules where cooperative learning was not used in the classroom. No significant differences were found between the experimental and control groups by age, *t*_(507)_ = 1.206, *p* = 0.306, or by gender, χ2(1) = 1.495, *p* = 0.221.

It is important to note that the handbook for the undergraduate degrees in Preschool, Primary, and Social Education at the University of Extremadura contains numerous activities and content related to group work. Therefore, group work is very important for all the students who participated in the study.

### Instruments

The Academic Goals Questionnaire (AGQ) by Hayamizu et al. (1989), adapted by Hayamizu and Weiner (1991) and translated into Spanish by García et al. (1998), contains 20 items that explore the types of academic goals pursued by students. Answers are categorized on a scale ranging from 1 to 5, with 1 indicating “never” and 5 “always.” Drawing on Dweck’s theory (1986) of learning and performance goals, Hayamizu et al. (1989) developed a questionnaire based on three types of academic goals: learning goals and two performance goals, social reinforcement goals and achievement goals. The learning goals (8 items, e.g., “I study because I find solving problems interesting”) evaluate the extent to which students apply themselves to learning with the intention of acquiring knowledge and improving their skills. The social reinforcement goals (6 items, e.g., “I study because I want others to see how clever I am”) measure the tendency among students to study with the aim of obtaining approval and avoiding rejection from classmates, teachers and parents. The achievement goals (6 items, e.g., “I study because I want to get good grades”) assess students’ desire to score highly on their exams and be able to continue their studies. In our study, the questionnaire had a reliability (Cronbach’s alpha) of.848 for the learning goals, 0.760 for the social reinforcement goals and.772 for the achievement goals. In addition, a confirmatory analysis of the questionnaire showed that the three independent factor model proposed by the original authors had a good fit (χ2/df = 2.439; GFI = 0.919; IFI = 0.918; TLI = 0.902; CFI = 0.917; RMSR = 0.071; RMSEA = 0.057).

### Design

A quasi-experimental methodology with a pretest-posttest between-groups design and control group was used for this study. The participants were not selected at random as their study groups were already established and could not be randomly formed (Campbell and Stanley, 2005) because the aim was to preserve the authenticity of the classroom and the learning environment. The experimental group was made up of five sub-groups (first-year and second-year students on the undergraduate degrees in Preschool and Primary Education and first-year students on the undergraduate degree in Social Education), which were selected because of the use of cooperative learning techniques in the classroom. To ensure that all the experimental sub-groups were equivalent, groups of four students were assembled and cooperative techniques, were used on 6 occasions per module (9 h, 6 sessions of 1.5 h of duration) over six consecutive weeks at the rate of one per week, Requiring monitoring by the teacher and a high level of cooperation and interdependence between group members (e.g., jigsaw, 1-2-4). The groups were heterogeneous in terms of gender, age and academic level.

The control group did not receive any intervention and was made up of three groups of students (first-year Preschool Education, second-year Primary Education and second-year Social Education). The students in the control group carried out group work in the traditional manner: the teacher instructed the students to form groups (either at random or according to students’ affinities) to complete a task, which they worked on independently until they had finished. In this approach, interdependence, individual responsibility and communication skills were assumed or disregarded.

### Procedure

The project began with a period of training for participating teachers on cooperative learning in the first semester of the 2018-2019 academic year (CL approaches and techniques, teacher’s role in CL and evaluation). Particular emphasis was placed on the importance of regularly observing the groups’ interactions and progress, intervening when necessary to help the students move forward with the task, providing individual and group feedback on their performance and results and including time for reflection on what worked well in the group and what could be improved.

Before the questionnaire was administered, the students gave their written informed consent to participate in the study in line with the ethical guidelines established by the American Psychological Association (2009). The anonymity and confidentiality of the data and their use for research purposes only was guaranteed. All the experimental sub-groups and the control group were evaluated pre-intervention and post-intervention. The study was approved by The University of Extremadura Ethics Committee.

### Data Analysis

Firstly, in order to check the assumptions of normality and homoscedasticity, Kolmogorov-Smirnov and Levene tests were run on the data. All contrasts had *p* > 0.05, justifying the use of Student’s *t*-test for paired samples and analysis of covariance (ANCOVA). A reliability analysis was performed (Cronbach’s alpha), the factor structure of the AGQ was confirmed and effect size tests were applied (Cohen’s *d*, Hedges’ *g*, and η*^2^*). The statistical analysis was carried out using SPSS software (version 21).

## Results

First of all, between-group (control/experimental) and within-group (pretest-posttest) pretest comparisons were performed to ascertain whether the pretest scores on the AGQ provided a suitable basis for between-group (control/experimental) comparison, reducing the likelihood that estimates associated with the independent variable would be due to other factors not considered in this study, and to confirm the efficacy of the intervention. In addition, the effect size was calculated using Cohen’s *d* and Hedges’ *g* to supplement the information from the statistical significance tests (Student’s *t*-test) (Table 2).

**TABLE 2:** Within-group and between-group mean differences and effect sizes for the Academic Goals Questionnaire (AGQ), experimental and control groups.

	Experimental group (*N* = 320)							Control group (*N* = 121)							Control/experimental group					
	Pretest		Posttest		Within-group			Pretest		Posttest		Within-group			Between-group pretest			Between-group posttest		
								
AGQ	*M*	*SD*	*M*	*SD*	*t*	*p*	*d*	*M*	*SD*	*M*	*SD*	*t*	*p*	*d*	*t*	*p*	*g*	*t*	*p*	*g*
Learning goals	30.41	4.60	31.92	4.73	−7.558	<001	0.32	30.05	5.17	28.93	5.24	7.558	<001	0.22	−0.695	0.487	0.07	−5.925	<001	0.61
Social reinforcement goals	9.60	3.21	9.83	3.84	−1.360	0.175	0.06	9.04	3.18	10.17	3.43	−2.821	0.006	0.34	−1.677	0.094	0.18	0.826	0.386	0.09
Achievement goals	27.09	3.29	27.23	3.42	−0.753	0.452	0.04	26.76	3.52	26.08	3.46	4.532	<001	0.19	−0.922	0.357	0.10	−3.197	0.001	0.33

In the pretest comparisons between the control and experimental groups, the absence of significant differences (*p* ≤ 0.05) in the scores on the AGQ shows equivalence between groups as a basis for comparison (Table 2). Meanwhile, in the between-group (control/experimental) posttest comparisons, the experimental group obtained significantly higher scores (*p* < 0.001) on learning goals and achievement goals, with a medium effect size (Table 2).

To control for possible variations due to differences between the control group and the experimental group, Student’s *t*-test for paired samples was performed (Table 2) to ascertain whether or not the results could be attributed to the independent variable (intervention).

The comparisons within the experimental group displayed a significant increase (*p* < 0.001) between the pretest and posttest score for the learning goals factor, with a small effect size (*d* = 32). Within the control group, there was a significant decrease (*p* < 0.001) between the pretest and posttest scores for the learning goals and achievement goals factors, with a very small effect size (*d* ≤ 22), and a significant increase (*p* = 0.006) with a small effect size (*d* = 0.34) for the social reinforcement goals factor.

Finally, to eliminate the effect attributable to variables not included in the study design and not subject to experimental control from the dependent variables (AGQ posttest scores) and to supplement the within-group and between-group tests, between-subject effects tests (ANCOVA) were performed. The pretest scores for the dependent variables were used as covariates and the experimental/control groups were used as a fixed factor (Table 3).

**TABLE 3:** Between-subject effects test (ANCOVA).

AGQ posttest	Origin pretest	Type III sums of squares	*gl*	Quadratic mean	*F*	Sig.	η2
F1	F1. Learning goals	6029.279	1	6029.279	577.948	<0.001	0.568
	Intervention groups	702.482	1	702.482	67.338	<0.001	0.141
	Error	4579.743	439	10.432			
F2	F2. Social reinforcement goals	1506.631	1	1506.631	143.065	<0.001	0.245
	Intervention groups	38.557	1	38.557	3.661	0.056	0.008
	Error	4633.671	440	10.531			
F3	F3. Achievement goals	1987.783	1	1987.783	272.310	<0.001	0.381
	Intervention groups	80.332	1	80.332	11.005	0.001	0.024
	Error	3233.770	443	7.300			

*AGQ factors/Intervention group.*

The ANCOVA (Table 3) shows significant differences (*p* < 0.05) between the groups (experimental and control) for the learning goals factor, with a large effect size (η2 = 0.14), and the achievement goals factor, with a small effect size (η2 = 0.02). This confirms the results of the between-group comparisons (Table 3). Therefore, the differences observed between the pretest and posttest scores for learning goals and achievement goals among the experimental and control groups may be attributed to the intervention.

## Discussion

This study aimed to evaluate the impact of a cooperative learning programme on university students’ academic goals using an intervention based on cooperative techniques requiring high levels of responsibility and interdependence among group members.

With a view to fulfilling the study objectives and ensuring that our results could be generalized, the reliability and validity of the Academic Goals Questionnaire (AGQ) by Hayamizu et al. (1989) was first evaluated. The three factor model proposed in the original study of the questionnaire was an adequate fit for our data. The indices obtained optimal values, showing sufficient reliability and validity to allow the results to be generalized (Costello and Osborne, 2005).

One of the initial objectives of our analysis in this study was to conduct a between-group comparison (experimental and control groups) to check that the differences between the pretest and posttest scores owed to the intervention and not to other factors and to confirm the efficacy of the intervention. No significant differences were observed in the pretest scores for the experimental and control groups, indicating equivalence between groups as a basis for comparison. The posttest comparison showed that cooperative learning techniques led to significant changes to students’ learning goals and achievement goals, with medium to large effect sizes found for learning goals (*g* = 0.61). Education research tends to produce lower values than other disciplines. When innovative methodologies are applied, values between *d* = 0.30 and *d* = 0.33 are considered relevant (Borg et al., 1993; Valentine and Cooper, 2003). Hattie (2009) finds a mean effect size of *d* = 0.40 in an analysis of 5,00,000 interventions in educational settings.

Secondly, pretest-posttest comparisons within the experimental group show the efficacy of cooperative learning for learning goals (*d* = 0.32). Given that the within-group comparisons (ANCOVA) show significant changes in achievement goals and especially in learning goals, and that within-group comparisons in the experimental group only show changes in learning goals, it is possible to conclude that the intervention using cooperative learning techniques primarily influenced university students’ learning goals.

Why did team work using cooperative learning techniques improve learning goals? Cooperative learning has considerable educational benefits, including intrinsic motivation, positive attitudes toward the subject, improved self-esteem, social support, group cohesion and participation (Fernandez-Rio et al., 2017; Han and Son, 2020; Cecchini et al., 2021; Liu and Lipowski, 2021). Generally speaking, cooperative learning environments tend to be more dynamic, appealing and enjoyable, as well as giving students more responsibility and power over their learning, enhancing perceived autonomy and competence and making a significant contribution to improving learning goals (Slavin, 1983; Johnson and Johnson, 1990; Amin, 2020; Han and Son, 2020).

On the other hand, the techniques used (e.g., jigsaw, 1-2-4) encourage high levels of interdependence and responsibility among group members. In cooperative learning situations, students become aware that they depend on one another and must push themselves to do their best. The team members share equal responsibility for their learning. Students consider that their team has worked more effectively when they perceive there to be responsibility within the team (León del Barco et al., 2017, 2018). Each team member undertakes to carry out their share of the work and the team is seen as being responsible for achieving the objectives. These mechanisms of interdependence and responsibility in particular enhance students’ overall motivation and improve learning goals.

A number of studies have demonstrated a direct relationship between responsibility and intrinsic motivation (Belando et al., 2015; Menéndez and Fernández-Rio, 2017; Méndez-Giménez et al., 2018; Fernández-Espínola et al., 2020; Fuertes et al., 2020) and self-determined motivation (Merino-Barrero et al., 2019). Based on these existing studies and on our own results, it is clear that responsibility in cooperative learning techniques plays a central role in encouraging students to focus their goals on learning.

These changes were not observed in the control group, whose posttest scores were lower than their pretest scores in two factors on the Academic Goals Questionnaire (AGQ) and increased with significant differences in the social reinforcement goals factor. The students in the control group carried out group work in the traditional manner, with interdependence and individual responsibility assumed or not taken into consideration. In situations where responsibility for learning is not shared and students do not apply themselves in full, learning tasks are tackled for academic reasons but also for prosocial reasons. Studies such as Schneider et al. (1996) corroborate the importance of social goals and of the search for approval from others in particular. For Valle et al. (2006), as well as goals oriented toward learning, other goals such as the quest for positive social status and approval from others encourage engagement in learning activities.

Therefore, our results confirm that motivation for learning would depend on the context in which interpersonal interaction occurs (Johnson and Johnson, 2005). In such a way that, if learning occurs in a context of positive interdependence in which students support, help, and encourage effort, the intrinsic motivation toward learning will be greater, and when there is no interdependence, or if this is minimal, the lower the learning motivation and the higher the achievement motivation.

### Limitations

The limitations of this study include those inherent to quasi-experimental designs in general, which are linked to working with natural groups and being unable to assign participants to experimental and control groups at random. This means that the experimental variables cannot be fully controlled and the results should be interpreted with caution. Furthermore, Generalization of the results for the masculine population is difficult, because the majority of the students enrolled are female. Another limitation of this study relates to monitoring changes and measuring the impact of the programme over a longer period, taking different measures to evaluate whether or not the changes persisted over time. The sole use of self-report tests to collect information is another limitation, as they tend to produce more subjective results and may give rise to issues with social desirability bias. However, in spite of these limitations, this type of research is very useful as it provides information from real situations without artificial interference.

## Conclusion

Academic goals influence students’ behavior in the classroom and drive them to achieve a series of objectives in their academic lives (De la Fuente, 2004; Rodríguez and López, 2020). Goals are perhaps one of the most important personal variables affecting learning. Following Alonso and Montero (2001), if we know that learning goals are shaped by the motivation for competence (studying because you want to know more and gain greater knowledge), the motivation for control (learning autonomously, being responsible for your own learning) and intrinsic motivation (enjoying learning and educational activities), we can create the necessary conditions to boost these factors in the classroom. This study has shown that one of the best ways of doing this is by using cooperative learning techniques, which are dynamic and increase students’ perceptions of autonomy and competence, helping to boost motivation for competence, intrinsic motivation, and motivation for control in particular.

This is the first study to analyze the association between cooperative learning and academic goals. Our findings contribute new knowledge to the conceptual framework on cooperative learning, emphasizing the role of cooperative learning techniques in shaping learning goals. The main practical implication for university teaching staff is the importance of using cooperative learning techniques in the classroom.

According to Chickering and Gamson (1987), encouraging cooperation between students is one of the seven basic principles that should underpin university teaching staff’s training in order to guarantee optimum learning among university students. The importance of cooperative learning in university classrooms is justified not only by the specific functions of university teaching but also by the need for alternatives to more authoritarian, individualistic methodologies, which lead to gaps in students’ education, insecurity in problem-solving, minimal participation and poor critical thinking and reflection skills.

## Data Availability Statement

The original contributions presented in the study are included in the article/supplementary material, further inquiries can be directed to the corresponding author.

## Ethics Statement

The studies involving human participants were reviewed and approved by the University of Extremadura Ethics Committee. The patients/participants provided their written informed consent to participate in this study.

## Author Contributions

SM-L, BL-d-B, M-IP-d-R, and VL-R: conception and design of the work and drafting the work. SM-L and BL-d-B: analysis and interpretation of the data. All authors contributed to the article and approved the submitted version.

## Conflict of Interest

The authors declare that the research was conducted in the absence of any commercial or financial relationships that could be construed as a potential conflict of interest.

## Publisher’s Note

All claims expressed in this article are solely those of the authors and do not necessarily represent those of their affiliated organizations, or those of the publisher, the editors and the reviewers. Any product that may be evaluated in this article, or claim that may be made by its manufacturer, is not guaranteed or endorsed by the publisher.
